# Supplementary material for: Yersinia deploys type III-secreted effectors to evade caspase-4 inflammasome activation in human cells
Source: mBio. 2023 Aug 24;14(5):e01310-23. doi: 10.1128/mbio.01310-23 (PMC10653943; doi:10.1128/mbio.01310-23)

Table S1. *Yersinia* strains used in this study

Strain name	Relevant characteristics	Reference or Source
IP2666 (WT *Yptb*)	Wild-type, pYV^{+}, naturally *yopT*$^{-}$	(130)
Δ6 Yptb	*yopEHJMKO*$^{-}$	(79)
ΔyopE Yptb	*yopE-*	(102)
ΔyopH Yptb	*yopH-*	(105)
ΔyopM Yptb	*yopM-*	This study and (40)
ΔyopJ Yptb	*yopJ-*	(79)
ΔyopK Yptb	*yopK-*	(45)
ΔyopO Yptb	*yopO-*	(128)
ΔyopEH Yptb	*yopEH-*	(105)
ΔyopEK Yptb	*yopEK-*	This study and (129)
ΔyopHK Yptb	*yopHK-*	This study and (129)
ΔyopEHK Yptb	*yopEHK-*	This study and (129)
ΔyopEHΔinv Yptb	*yopEH-* invasin-	This study and (129)
ΔyopEHΔyadA Yptb	*yopEH-* YadA-	This study and (70)
ΔyopEHΔinvΔyadA Yptb	*yopEH-* invasin- YadA-	This study and (70, 119)
Δ6Δinv Yptb	*yopEHJMKO-* invasin-	This study and (119)
Δ6ΔyadA Yptb	*yopEHJMKO-* YadA-	This study and (70)
Δ6ΔinvΔyadA Yptb	*yopEHJMKO-* invasin- YadA-	This study and (70, 119)

Figure S1 (related to Fig. 1). *Yersinia* Yops evade inflammasome activation. (A, E) THP-1 macrophages were infected with PBS (Mock) or WT *Yptb* or Δ*6 Yptb*. (A) Percent cytotoxicity normalized to cells treated with 2% triton and (E) release of IL-1β into the supernatant was measured by ELISA at 6hpi. (B) Polarized Caco-2 cells were infected with PBS or WT *Yptb* or Δ*6 Yptb*. Release of IL-18 into the apical and basolateral supernatant was measured by ELISA at 6hpi. (C) WT Caco-2 cells were infected with the indicated MOI and strain of *Yptb*. IL-18 release was measured by ELISA at 6hpi. (D) WT Caco-2 cells were infected with PBS (Mock) or WT *Yptb* or Δ*6 Yptb*. Release of IL-1β into the supernatant was measured by ELISA at 6hpi. ** $p < 0.01$, *** $p < 0.001$, **** $p < 0.0001$ by one-way ANOVA. Shown are averages and error bars representing the standard deviation from at least three pooled experiments.

Figure S2 (related to Fig. 2). Caspase-1 and caspase-8 are not absolutely required for Δ*6 Yptb*-induced inflammasome activation in human IECs. (A) WT Caco-2 cells or two independent single cell clones of *CASP1*$^{-/-}$ Caco-2 cells were infected with PBS (Mock), WT *Yptb*, or Δ*6 Yptb*. (B) One hour prior to infection, WT Caco-2 cells were treated with 20 μM YVAD or DMSO as a vehicle control. Cells were then infected with PBS (Mock), WT *Yptb* or Δ*6 Yptb*. (A, B) IL-18 release into the supernatant was measured by ELISA at 6hpi. (C) Knockdown efficiency of siRNA targeting *CASP8* in WT Caco-2 cells was measured by qRT-PCR and normalized to housekeeping gene *HPRT* and calculated relative to control-siRNA-treated cells. (D) WT Caco-2 cells were treated with siRNA targeting a control scrambled siRNA or siRNA targeting *CASP8* for 72 hours. (E) One hour prior to infection WT Caco-2 cells were treated with 20 μM IETD or DMSO as a vehicle control. (D, E) Cells were infected with PBS (Mock), WT *Yptb* or Δ*6 Yptb*. IL-18 release into the supernatant was measured at 6hpi. (F, G) WT or two independent single cell clones of *CASP1*$^{-/-}$ Caco-2 cells were treated with siRNA targeting a control scrambled siRNA or siRNA targeting *CASP8* for 72 hours. (F) IL-18 release into the supernatant was measured at

6hpi. (G) Knockdown efficiency of siRNA targeting *CASP8* in WT Caco-2 cells was measured by qRT-PCR and normalized to housekeeping gene *HPRT* and calculated relative to control-siRNA-treated cells. *** $p < 0.001$ by two-way ANOVA. (A-E) Shown are averages and error bars representing the standard deviation from at least three pooled experiments. (F, G) Error bars represent standard deviation of triplicate wells and are representative of two independent experiments.

Figure S3 (related to Fig. 2). Caspase-4 contributes to Δ*6 Yptb*-induced inflammasome activation in human macrophages. (A) WT or two independent single cell clones of *CASP4*$^{-/-}$ THP-1 macrophages were infected with PBS (Mock), WT *Yptb* or Δ*6 Yptb*. (B) One hour prior to infection WT THP-1 macrophages were treated with 20 μM ZVAD or DMSO as a vehicle control. (A, B) IL-1β release into the supernatant was measured at 6hpi. (C) Knockdown efficiency of siRNA targeting *CASP5* in WT Caco-2 cells was measured by qRT-PCR, normalized to *HPRT* and calculated relative to control-siRNA-treated cells. (D) WT Caco-2 cells were treated with siRNA targeting a control scrambled siRNA or siRNA targeting *CASP5* for 72 hours. IL-18 release was measured at 6hpi. ** $p < 0.01$, *** $p < 0.001$ by two-way ANOVA. Shown are averages and error bars representing the standard deviation from at least three pooled experiments.

Figure S4 (related to Fig. 3). NLRP3, NAIP/NLRC4 and ASC are dispensable for *Δ6 Yptb*-induced inflammasome activation in human IECs. (A) One hour prior to infection cells were treated with 10 μM MCC950 or DMSO as a vehicle control. Cells were then infected with PBS (Mock) WT *Yptb* or Δ*6 Yptb.* (B, C) WT or two independent single cell clones of (B) *NAIP*$^{-/-}$ or (C) *PYCARD*$^{-/-}$ Caco-2 cells were infected with PBS (Mock), WT *Yptb*, or Δ*6 Yptb*. (A, B, C) IL-18 release was measured in supernatants at 6hpi. Shown are averages and error bars representing the standard deviation from at least three pooled experiments.

Figure S5 (related to Fig. 5). YopE and YopH block internalization in human cells. (A) WT Caco-2 cells or (B) WT THP-1 macrophages were infected with the indicated strain of *Yptb* at an MOI of 20 and lysed at 2 hpi. Bacteria were plated on *Yersinia*-selective agar to enumerate CFUs. (C, D) WT Caco-2 cells were seeded on glass coverslips and infected with indicated strain of *Yptb* expressing GFP at an MOI of 20. Cells were fixed at 2hpi and stained for extracellular *Yptb* (Red) and DAPI to label DNA (blue). (C) Representative images are shown. Scale bar represents 10 μm. (D) Proportion of total bacteria that was intracellular (green only) was scored unblinded by fluorescence microscopy. Shown are averages and error bars of coverslips from independent experiments with 20 fields scored per coverslip. (E, F) One hour prior to infection, WT Caco-2 cells were treated with 10 μM cytochalasin D or DMSO as a vehicle control. Cells were then infected with PBS (Mock) or indicated strain of *Yptb* at an MOI of 20 (E) or 60 (F). (E) Cells were lysed at 2hpi and bacteria were plated on *Yersinia*-selective agar to calculate CFUs. (F) Release of IL-18 into the supernatant was measured at 6hpi. * $p < 0.05$, *** $p < 0.001$, **** $p < 0.0001$ by one-way ANOVA (A, B, D) or two-way ANOVA (E, F). Shown are averages and error bars representing the standard deviation from at least three pooled experiments.

Figure S6 (related to Fig. 5). (A, B) One hour prior to infection, WT Caco-2 cells were treated with 25 μM Akt inhibitor VIII or DMSO as a vehicle control. Cells were then infected with PBS (Mock) or indicated strain of *Yptb* at an MOI of 20 (A) or 60 (B). (A) Cells were lysed at 2hpi and bacteria were plated on *Yersinia*-selected agar to calculate CFUs. (B) Release of IL-18 into the supernatant was measured at 6hpi. (C, E) WT Caco-2 cells were treated with a control scrambled siRNA or siRNA targeting *ITGA5* for 72 hours. Cells were then infected with PBS (Mock) or indicated strain of *Yptb* at an MOI of 20 (C) or 60 (D). (C) Cells were lysed at 2hpi and

bacteria were plated on *Yersinia*-selected agar to calculate CFUs. (D) Release of IL-18 into the supernatant was measured at 6hpi. (E) Knockdown efficiency of siRNA targeting *ITGA5* in WT Caco-2 cells was measured by qRT-PCR and normalized to housekeeping gene *HPRT* and calculated relative to control-siRNA-treated cells. (F, G) WT Caco-2 cells were infected with PBS (Mock) or indicated strain of *Yptb* at an MOI of 20 (F) or 60 (G). (F) Cells were lysed at 2hpi and bacteria were plated on *Yersinia*-selected agar to calculate CFUs. (G) Release of IL-18 into the supernatant was measured at 6hpi. Shown are averages and error bars representing the standard deviation from at least three pooled experiments.

Figure S1

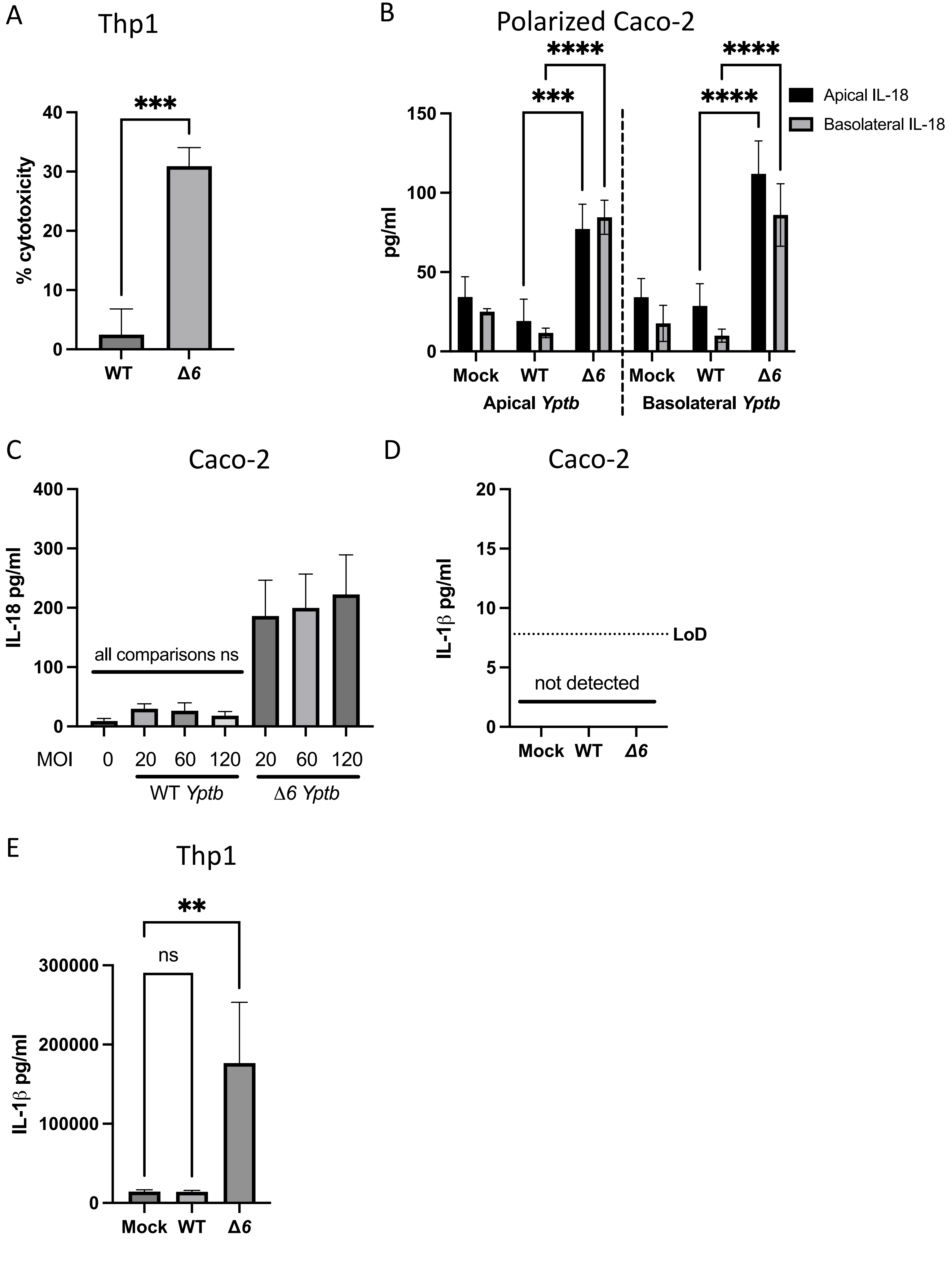

A
Thp1
% cytotoxicity
0
10
20
30
40
***
WT
Δ6
B
Polarized Caco-2
pg/ml
0
50
100
150
***
****
****
****
Apical IL-18
Basolateral IL-18
Mock
WT
Δ6
Mock
WT
Δ6
Apical Yptb
Basolateral Yptb
C
Caco-2
IL-18 pg/ml
0
100
200
300
400
all comparisons ns
MOI
0
20
60
120
20
60
120
WT Yptb
Δ6 Yptb
D
Caco-2
IL-1β pg/ml
0
5
10
15
20
LoD
not detected
Mock
WT
Δ6
E
Thp1
IL-1β pg/ml
0
100000
200000
300000
**
ns
Mock
WT
Δ6

Figure S2

A

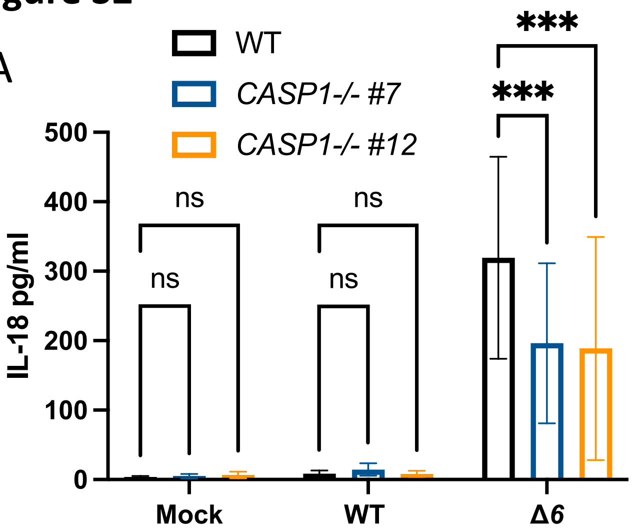

B

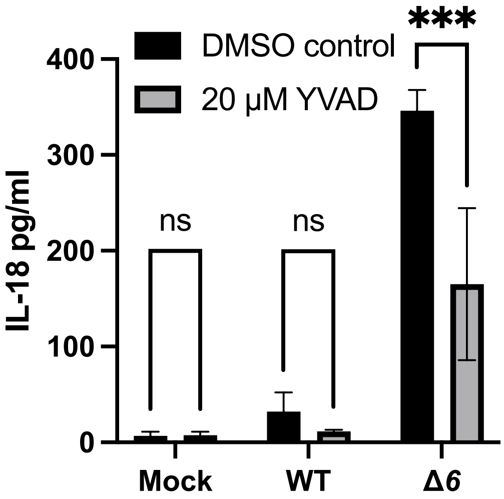

C

Average knockdown efficiency

	CASP8
WT	66%

D

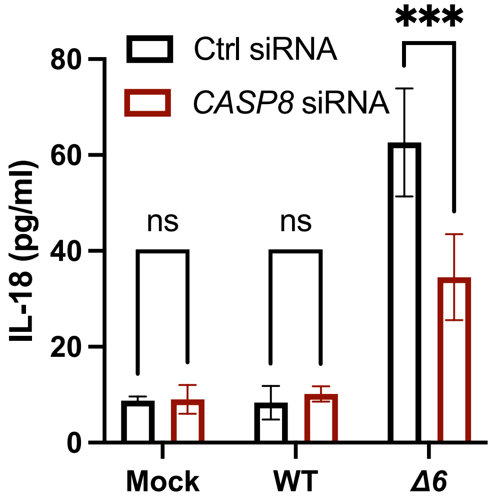

E

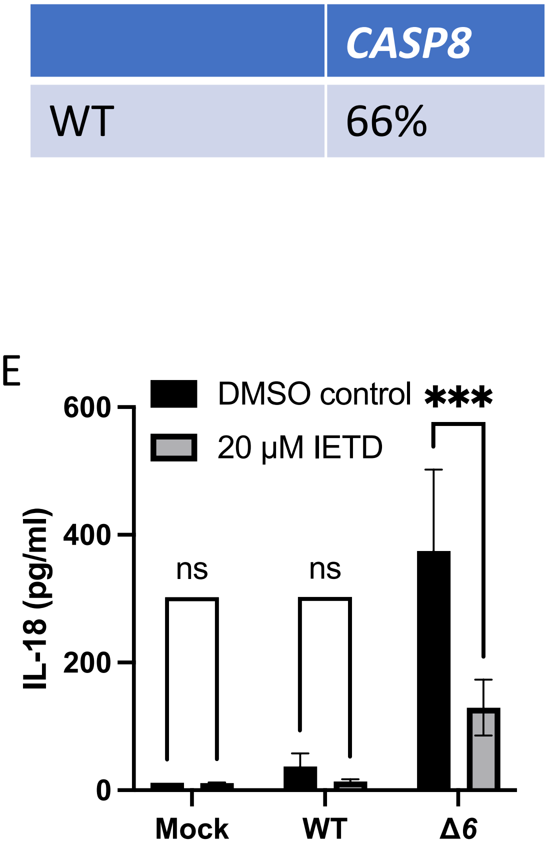

F

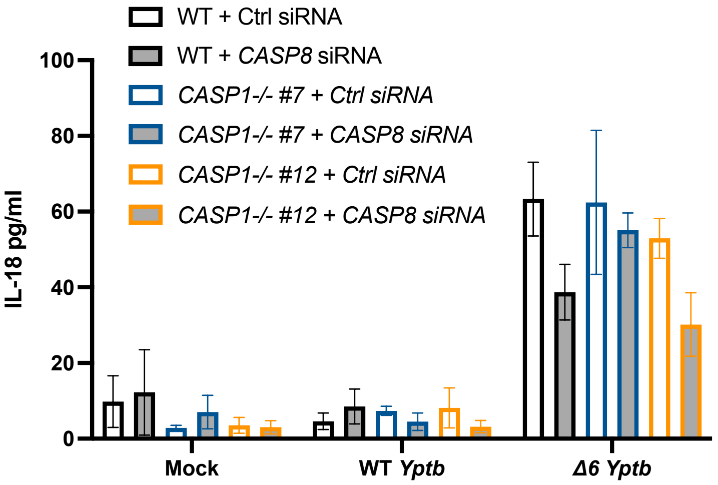

G

Average knockdown efficiency

	CASP8
WT	57%
CASP1-/- #7	68%
CASP1-/- #12	74%

Figure S3

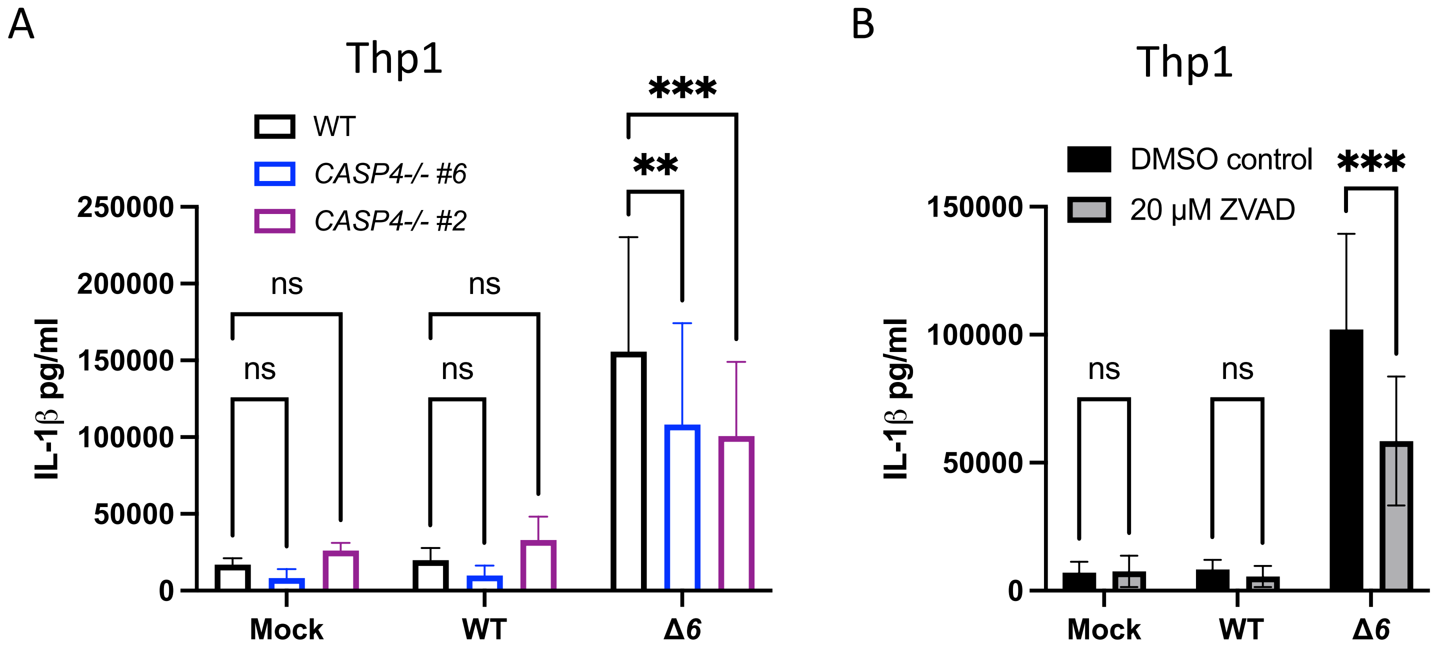

C

Caco-2

Average knockdown efficiency

	CASP5
WT Caco-2s	75%

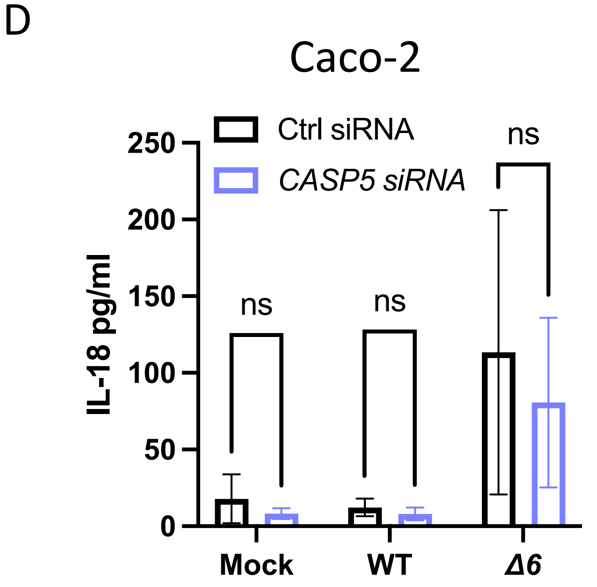

Figure S4

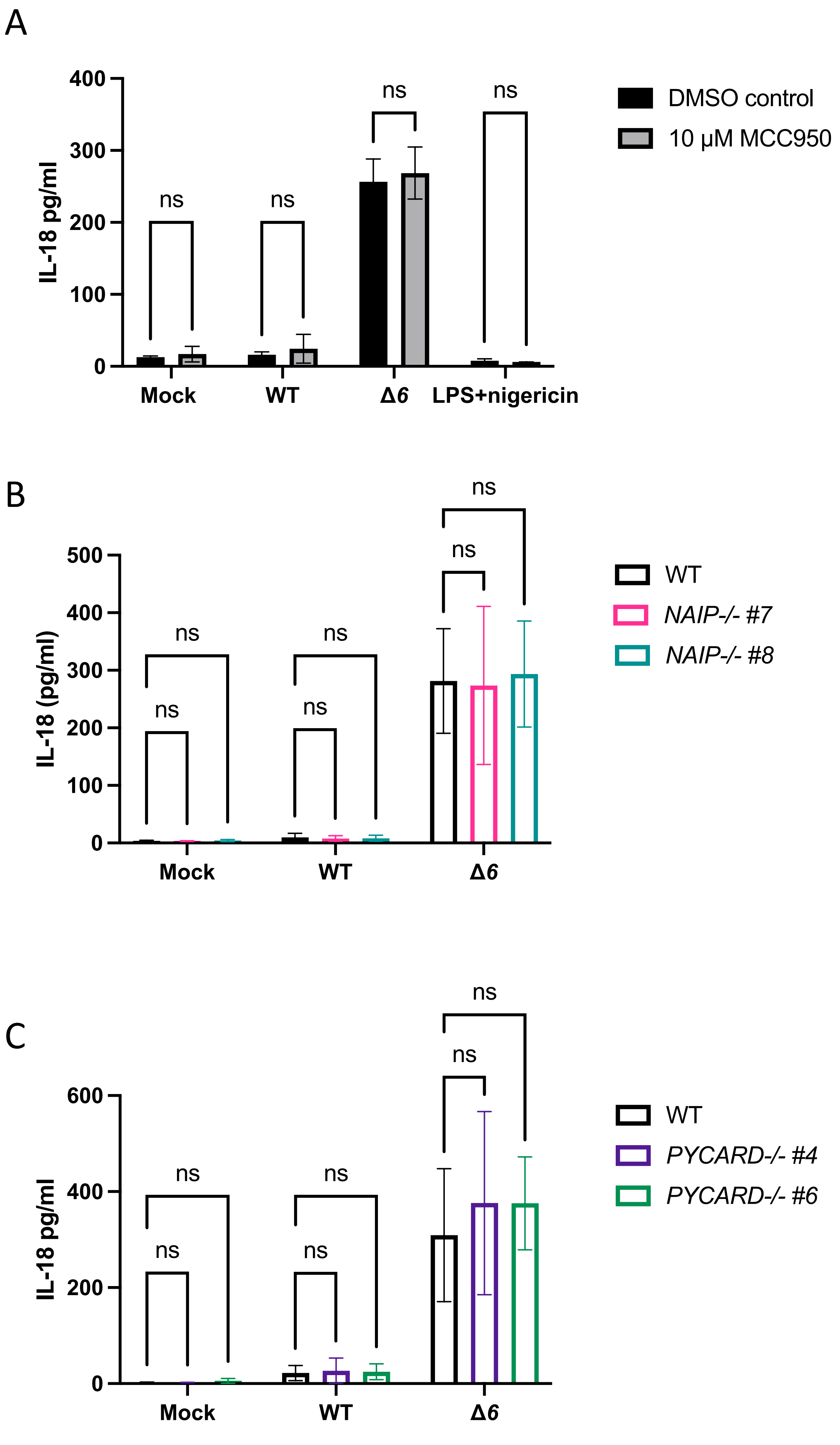
A
IL-18 pg/ml
400
300
200
100
0
ns
ns
ns
ns
Mock
WT
Δ6
LPS+nigericin
DMSO control
10 μM MCC950
B
IL-18 (pg/ml)
500
400
300
200
100
0
ns
ns
ns
ns
ns
ns
Mock
WT
Δ6
WT
NAIP-/- #7
NAIP-/- #8
C
IL-18 pg/ml
600
400
200
0
ns
ns
ns
ns
ns
ns
Mock
WT
Δ6
WT
PYCARD-/- #4
PYCARD-/- #6

Figure S5

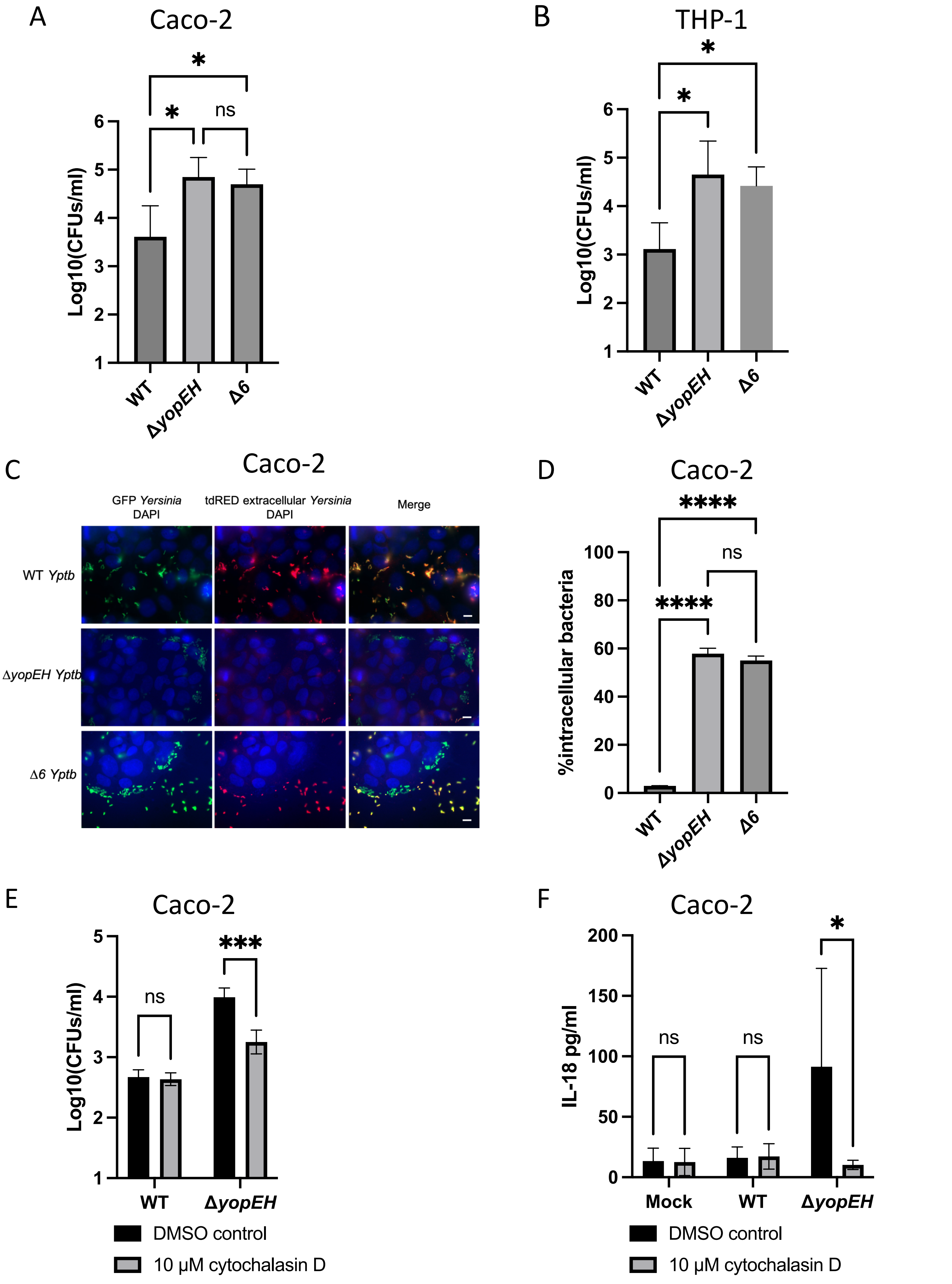
A
Caco-2
Log10(CFUs/ml)
WT
ΔyopEH
Δ6
ns
B
THP-1
C
Caco-2
GFP Yersinia DAPI
tdRED extracellular Yersinia DAPI
Merge
WT Yptb
ΔyopEH Yptb
Δ6 Yptb
D
%intracellular bacteria
E
F
IL-18 pg/ml
Mock
DMSO control
10 μM cytochalasin D

Figure S6

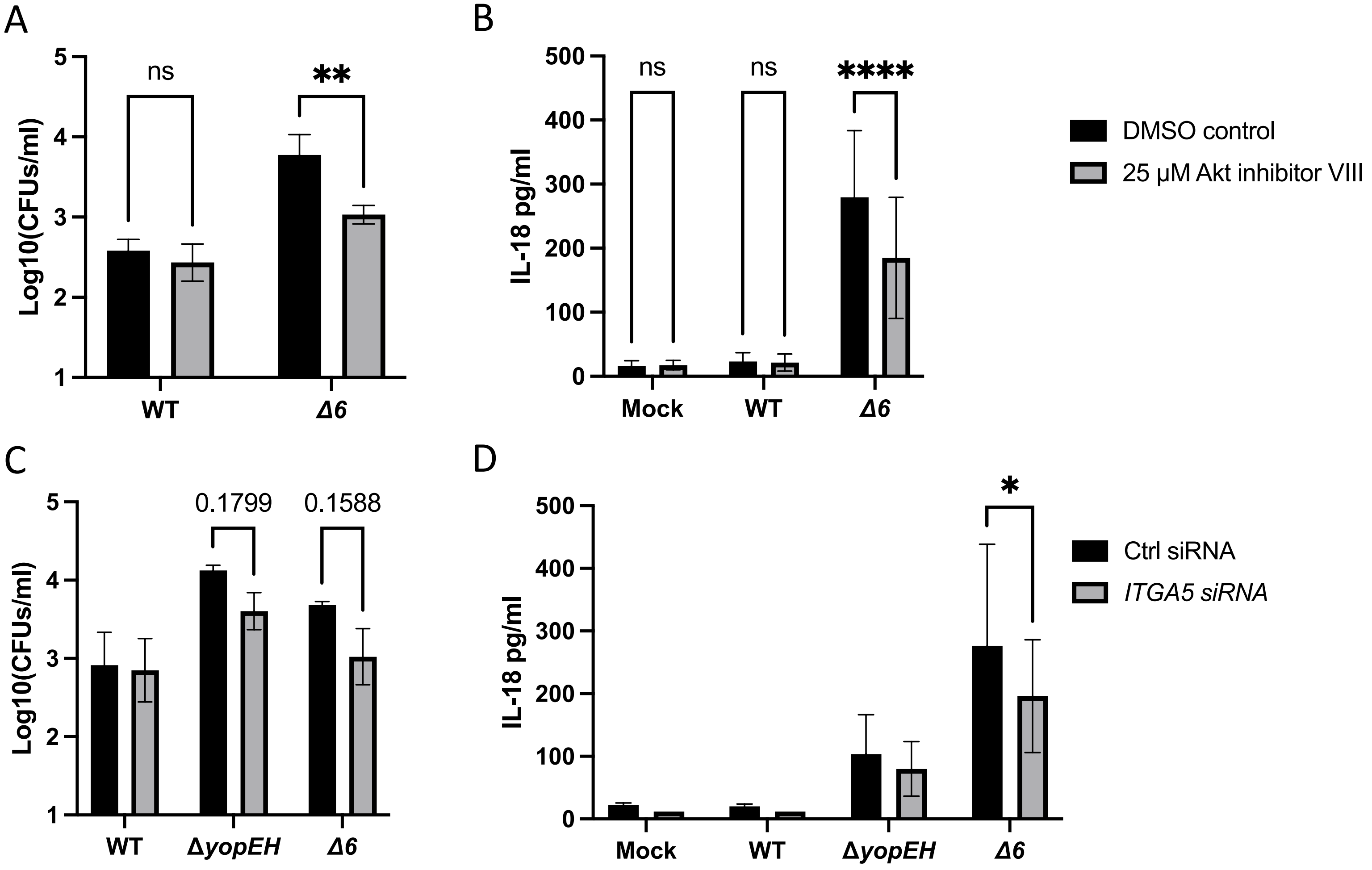

E

Average knockdown efficiency

	ITGA5
WT	65%

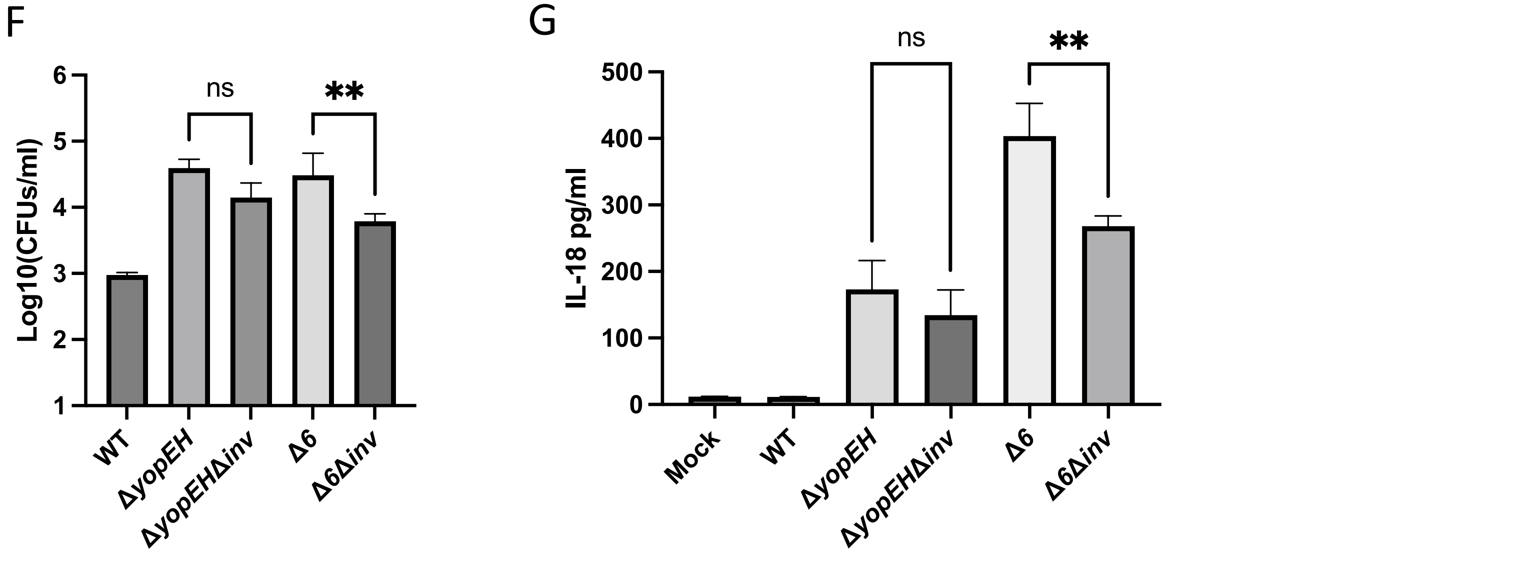

Supplement: Supplemental material — Table S1; Fig. S1 to S6. [file mbio.01310-23-s0001.pdf]
